# Production of Vitamin K2 Enriched Dairy Cream by *Bacillus subtilis Natto* Fermentation

**DOI:** 10.1002/fsn3.72119

**Published:** 2026-07-17

**Authors:** Hasan Moharampour, Sodeif Azadmard‐Damirchi, Javad Hesari, Mohammad Reza Afshar Mogaddam, Safar Farjnia

**Affiliations:** ^1^ Department of Food Science and Technology, Faculty of Agriculture University of Tabriz Tabriz Iran; ^2^ Food and Drug Safety Research Center Tabriz University of Medical Sciences Tabriz Iran; ^3^ Research Center of New Material and Green Chemistry Khazar University Baku Azerbaijan; ^4^ Drug Applied Research Center Tabriz University of Medical Sciences Tabriz Iran

**Keywords:** *Bacillus subtilis natto*, cream, fermentation, nattokinase, vitamin K_2_ (menaquinone‐4 menaquinone‐7)

## Abstract

Foods containing suitable amounts of vitamin K2 and nattokinase have several positive health effective properties. Therefore, in the present research, dairy cream was used as a widely consumed food product for fermentation with *Bacillus subtilis natto* (BSN) to introduce a new dairy product to the consumers. For this purpose, pasteurized dairy cream (30% fat) was inoculated with BSN and fermented at different temperatures (30°C and 37°C) and incubation times (12, 24, and 36 h) before being stored at refrigeration temperature (4°C). The fermented cream samples were evaluated for protein, ammonium content, protease activity, pH, acidity, nattokinase activity, vitamin K2 (menaquinone‐4, menaquinone‐7), and total microbial count during the process. The optimal fermentation conditions were found to be at 30°C for 24 h, during which the content of menaquinone‐4 (MK‐4) and menaquinone‐7 (MK‐7) increased from 10.92 to 142.27 ng/g and from 4.10 to 43.93 ng/g, respectively. Moreover, fermentation of cream with BSN led to a significant reduction in free ammonium (from 186.84 to 128.36 mg/100 g), and a pronounced increase in protease activity (from 0.003 to 0.328) in the optimum sample. Additionally, all cream samples inoculated with BSN showed a significant reduction (*p* < 0.05) in total microbial counts throughout the storage period. Sensory evaluation revealed that fermentation did not cause any undesirable properties in the prepared samples compared to the control sample. These findings demonstrate that BSN fermentation is an effective strategy to convert plain dairy cream into a functional product enriched with vitamin K2 and nattokinase, offering a promising innovation for the dairy industry.

## Graphical abstract

In the present research, dairy cream was used as a widely consumed food product for fermentation with *Bacillus subtilis natto* (BSN) to introduce a new dairy product to the consumers. For this purpose, pasteurized dairy cream (30% fat) was inoculated with BSN and fermented at different temperatures (30°C and 37°C) and incubation times (12, 24, and 36 h) before being stored at refrigeration temperature (4°C). The application of BSN in the production of fermented products revealed that this approach can be applied to produce dairy products with a high content of vitamin K2, enhanced nattokinase activity, cream stability, and texture.

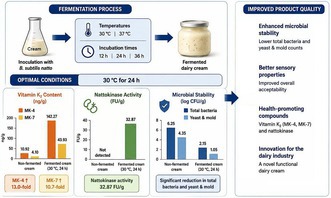

## 1 Introduction

Milk cream plays a significant role in the human diet and is commonly used in the production of various dairy products. However, its high levels of saturated fatty acids and cholesterol have raised concerns about its impact on health. As a result, efforts have been made to improve its nutritional profile through methods such as blending with unsaturated oils, fractionation, or using cholesterol adsorbents such as beta‐cyclodextrins (Kolarič et al. 2022). One innovative approach to addressing these concerns is fermentation, which not only preserves food but also produces health‐promoting metabolites without compromising sensory quality.

Fermentation is a traditional and sustainable biotechnological process that enhances the nutritional and functional properties of foods by generating bioactive compounds, improving microbial stability, and modifying texture and flavor (Adetuyi et al. 2025). Fermented foods have gained recognition for their positive impact on gut microbiota composition and overall health (Freitas et al. 2026). In dairy products, fermentation has been successfully used to introduce beneficial microorganisms and their metabolites. However, the choice of microbial culture is crucial in determining the final functional properties of the fermented product.

Among various fermented foods, natto—a traditional Japanese product made from soybeans fermented with *Bacillus subtilis natto* (BSN)—is particularly notable for its high content of the fibrinolytic enzyme nattokinase and the fat‐soluble vitamin K2 (menaquinone‐7, MK‐7). Fermentation with Bacillus species has been shown to modify the structure of food matrices and enhance their bioactive properties (Yu et al. 2025). These compounds have been associated with cardiovascular protection, osteoporosis prevention, and neuroprotective effects (Shih et al. 2010; Afzaal et al. 2022). Despite its high MK‐7 content, natto has a strong odor and flavor that may affect its consumption (Berenjian et al. 2015).

Previous studies have demonstrated that BSN can effectively produce menaquinones in various fermentation media, including soybean‐based substrates (Mahdinia et al. 2018) and optimized culture broths (Zhang et al. 2025). Additionally, recent research has shown that starter cultures can increase vitamin K2 content in dairy products such as different cheeses (Altuncu et al. 2024). However, to the best of our knowledge, these studies have focused either on nondairy matrices or on conventional dairy starter cultures, leaving a clear gap in the application of BSN specifically in dairy cream fermentation. Therefore, the aim of the present study was to ferment pasteurized dairy cream (30% fat) with BSN under different temperatures (30°C and 37°C) and incubation times (12, 24, and 36 h) to evaluate the resulting physicochemical, functional, microbial, and sensory properties of the fermented product.

## 2 Materials and Methods

### 2.1 Materials

Natto Starter Spores and pasteurized cream received from TAKAHASHI NATTOMOTO Co. (Japan) and Shirin Asal Food Industries (Tabriz, Iran), respectively. All chemicals and reagents listed were of analytical or HPLC grade. Folin–Ciocalteu reagent, 2,3‐dihydrophylloquinone, nutrient broth, menaquinone‐7 (MK‐7) standard, menaquinone‐4 (MK‐4) standard were purchased from Sigma‐Aldrich (USA). Sodium hydroxide, phosphotungstic acid, *n*‐butanol, ammonium standard solution, lactic acid, potassium chloride, boric acid, trichloroacetic acid, *n‐*hexane, methanol, dichloromethane, zinc chloride, acetic acid, sodium acetate, and calcium chloride were purchased from Merck (Germany).

### 2.2 Preparation of Inoculum

Commercial Natto Starter (10 g) was introduced to a 250 mL Erlenmeyer flask containing 200 mL of sterile nutrient broth and incubated at a temperature of 40°C for a period of 16 h on a rotary shaker at a speed of 200 rpm. Cell growth was measured at an optical density of 660 nm; after 16 h of incubation, the OD660 reached around 1.5 (10^7^–10^8^ CFU/mL). The culture was subjected to centrifugation at 12,000 rpm for 25 min, and the resulting cell pellet was diluted in 20 mL of Butterfield phosphate buffer to prepare the inoculum suspension (Wei et al. 2006).

### 2.3 Cream Production

Pasteurized cream with 30% fat content was prepared from Shirin Asl Food Industry Co. (Iran) and inoculated at 30°C and 37°C for 12, 24, or 36 h. The fermented cream was then kept at 4°C before the analysis (Bolling et al. 2005).

### 2.4 Determination of Protein Content

Total protein content was measured by determination of nitrogen content with the Kjeldahl method (Prandi et al. 2023). The protein content was computed by multiplying the nitrogen content by the factor 6.25.

### 2.5 Determination of Ammonia (NH₄^+^) Content

The concentration of ammonia in the fermented cream sample was determined by utilizing a modified AOAC colorimetric method (AOAC 25/973, 1995). A brominating reagent was made by taking 10 mL of 50% NaOH (in CO_2_‐free water) and mixing it with 90 mL of double‐distilled water and 1 mL of concentrated bromine. The mixing of sodium hydroxide, fresh thymol, and the bromine solution produced a color of the intensity related to ammonia content in a 2.5% phosphotungstic acid solution (180 mL for 20 g cream sample). n‐butanol was used for the separation of the clear color solution and the turbid portion. The color solution's absorption was checked with a spectrophotometer at a wavelength of 580 nm. The ammonia concentration (mg/100 g) was calculated from an ammonium standard curve (Wei et al. 2006).

### 2.6 Protease Activity Assay

The proteolytic activity at pH 6 was determined using the casein digestion method (Wu et al. 2013). A mixture of 1 mL of enzyme solution and 5 mL of 1.2% casein in 0.05 M phosphate buffer (pH 6) was mixed and kept at 35°C for 10 min. The reaction was terminated by adding 5 mL of 0.44 M trichloroacetic acid, and the mixture was filtered. To 2 mL of filtrate, 5 mL of 0.28 N NaOH, and 1.5 mL of Folin–Ciocalteu reagent (diluted 1:2 with water) were added. After incubating at 35°C for 15 min and measuring at 660 nm, the absorbance was recorded in the table.

### 2.7 pH Measurement

Three grams of fermented cream sample were mixed with 1.8 mL double‐distilled water, and pH was measured with a digital pH meter (Aydogdu et al. 2023).

### 2.8 Acidity Measurement

Cream samples (10 g) were diluted with 10 mL of distilled water and completely mixed. The resulting suspension was then filtered through Whatman filter paper, and 10 mL of the filtrate was titrated with 0.1 N NaOH along with phenolphthalein until the endpoint of a light pink color was observed and it became persistent. The treatable acidity was determined as lactic acid percentage by the given formula (Lorieau et al. 2018).

$$\text{Acidity}\;\left(\%\right)=\left(V\times N\times 90.08\times 100\right)/W$$

where V = volume of NaOH consumed (mL), *N* = normality of NaOH, W = weight of sample (g).

### 2.9 Measurement of Nattokinase Activity

Initially, 1.4 mL of 50 mM KCl–H_3_BO_3_ buffer (pH 8.0) plus 0.4 mL of 0.72% fibrinogen were mixed and preincubated at 37°C for a duration of 5 min. After that, 0.1 mL of thrombin (20 U/mL) was incorporated, and the entire mixture was kept at 37°C for another 10 min. Then, 0.1 mL of sample extract was added, and the total mixture was incubated at 37°C for 60 min. To terminate the reaction, 2 mL of 0.2 M trichloroacetic acid was added, and the mixture was then centrifuged at 12,000 rpm for 5 min. The absorbance of the supernatant was determined at 275 nm. The determination of nattokinase activity was done using the given formula (Lee et al. 2015).

$$\text{Activity}=\left[\left(A\_\text{blank}-A\_\text{sample}\right)/0.01\right]\times \left(1/60\right)\times \left(1/0.1\right)\times D$$

where A_blank = absorbance of control, A_sample = absorbance of sample, and D = dilution factor of the sample.

### 2.10 Determination of Menaquinone‐7 and Menaquinone‐4

#### 2.10.1 Extraction

A sample weight of 1 g was blended with 4 mL of 2‐propanol, 20 ng of 2,3‐dihydrophylloquinone (internal standard), and 2 mL of distilled water. The heating was done at a temperature of 60°C, and afterwards, the mixture was extracted using 8 mL of hexane. The hexane layer was then dried by evaporation, dissolved in 2 mL hexane, and purified via a Sep‐Pak silica cartridge (Sungur et al. 2021).

#### 2.10.2 HPLC Analysis

The samples, MK‐7 and MK‐4 standards (5–100 ng/mL), were analyzed on a Shimadzu LC‐20A system equipped with a C18 column (5 μm, 250 × 4.6 mm) at 30°C. The mobile phase included a mixture of methanol/dichloromethane (90:10 v/v) containing 2 mM ZnCl_2_, 1 mM acetic acid, and 1 mM sodium acetate, at a flow rate of 1 mL/min. MK‐7 was detected fluorometrically (λex = 243 nm, λem = 430 nm) after postcolumn electrochemical reduction (Sungur et al. 2021). The internal standard method was applied for quantification.

### 2.11 Microbial Assay

Total aerobic mesophilic bacteria were enumerated by spread‐plating appropriate dilutions on Plate Count Agar (Merck, Germany). Plates were incubated at 30°C for 72 h, and colonies were counted (Li et al. 2024).

### 2.12 Sensory Properties

Sensory properties were evaluated by ten trained panelists using a 9‐point hedonic scale. Only overall acceptance scores are reported (Dehghani Khiavi et al. 2025).

### 2.13 Statistical Analysis

A factorial experiment in a completely randomized block design was used for sensory data, and a factorial experiment in a completely randomized design was applied for physicochemical and microbial data. We used the least significant difference (LSD) test for comparison among the treatment means. All the analyses were performed using SPSS software (version 22).

## 3 Results and Discussion

### 3.1 Protein Content

The amount of protein in dairy cream is a key factor for determining the nutritional and technological quality of cream, and it can be affected by both microbial metabolism and the processing conditions (Polishchuk et al. 2020). In this study, all samples inoculated with BSN showed a slight increase in protein content compared to the control (Table 1), with Sample 2 (incubated at 30°C for 24 h) showing the highest value. However, generally, there were no significant (*p* < 0.05) differences among the fermented dairy creams.

**TABLE 1:** Protein, ammonium content and protease activity of creams inoculated with *Bacillus subtilis natto* and incubated in different temperatures and times.

Sample	Protein (%w/w)	Ammonium content (mg/100 g)	Protease activity (optical density at 660 nm)
Control	3.26a±0.1817^*^	186.84g±1.1730	0.0032a±0.0038
Sample 1 (incubated in 30°C for 12 h)	3.42ab±0.1329	142.01b±0.2596	0.2083e±0.0204
Sample 2 (incubated in 30°C for 24 h)	3.49b±0.1140	128.36a±2.0472	0.3283f±0.0187
Sample 3 (incubated in 30°C for 36 h)	3.42ab±0.0636	144.76c±1.1931	0.1730d±0.0182
Sample 4 (incubated in 37°C for 12 h)	3.36ab±0.1193	154.50e±1.5531	0.0760c±0.0105
Sample 5 (incubated in 37°C for 24 h)	3.34ab±0.1096	147.24d±0.3604	0.1502d±0.0154
Sample 6 (incubated in 37°C for 36 h)	3.35ab±0.0240	161.80f±0.8988	0.0333b±0.0188

^*^ Different lowercase letters show differences between samples according to the Duncan test (*p* < 0.05).

The increased protein formation at 30°C relative to the control can be considered a result of the balanced metabolic condition, where nitrogen sources (ammonium and free amino acids) have been mainly directed towards the synthesis of cellular protein and biomass. In contrast, at 37°C, more active proteolytic enzymes might cause the proteins to be partially broken down into peptides and free amino acids, some of which may not be detected by the colorimetric methods for measuring proteins (e.g., Bradford or BCA), leading to lower apparent protein values (Polishchuk et al. 2020).

Comparable behavior has been reported in natto fermentation systems, where BSN has been shown to effectively alter protein fractions via proteolysis and biomass synthesis, leading to changes in total and soluble protein contents during fermentation of soybean substrates. For instance, the studies related to natto fermentation describe increased soluble nitrogen, peptides, and microbial biomass protein (Weng and Chen 2010). These reports are in agreement with the present findings, and the conclusion that BSN can redirect nitrogen metabolism and protein partitioning in fermented matrices is supported.

### 3.2 Ammonia (NH_4_^+^) in Product

Ammonia (NH_4_^+^) in the product is considered one of the main indicators of bacterial metabolic activity and nitrogen usage during fermentation, as it reflects the equilibrium between nitrogen release and absorption (Zhou et al. 2021). In the present study, all samples inoculated with BSN exhibited significantly (*p* < 0.05) lower NH_4_^+^ levels than the control sample, while Sample 3 (incubated at 30°C for 36 h) showed the lowest ammonium content (Table 1). This steady reduction implies that BSN actively absorbs ammonium for biosynthetic processes under the studied conditions, thereby decreasing free ammonium in the cream matrix.

The observed decrease in ammonium in inoculated samples can be explained by the metabolic activities of BSN, which uses ammonium as a nitrogen source for the synthesis of amino acids, nucleic acids, and other cellular components. The microbial metabolism incorporates ammonium into organic nitrogen compounds, for example, peptides and amino acids, thus leading to a decrease in its free concentration in the medium. In addition, pH changes during fermentation influence the equilibrium between NH_4_^+^ and NH_3_; a decrease in pH can facilitate bacterial ammonium uptake (Gunka and Commichau 2012). Such metabolic changes have been noted in natto fermentation, where ammonium production and deamination reactions through glutamate dehydrogenase and other pathways contribute significantly to the ammonia balance during secondary fermentation by BSN (e.g., ammonia‐releasing reactions identified in natto systems) (Kada et al. 2008).

Moreover, fermentation temperature plays a crucial role in the regulation of metabolic activity and nitrogen consumption. High temperatures (e.g., 37°C), increased metabolic rates may cause increased nitrogen turnover overall and faster ammonium assimilation or conversion due to enhanced metabolic rates, whereas slower metabolism at lower temperatures can lead to different NH_4_^+^ dynamics. These findings support the idea that the fermentation reactions of ammonium content are tightly linked to both bacterial metabolic pathways and environmental conditions (Ju et al. 2019).

### 3.3 Protease Activity Assay

The activity of proteases is an essential characteristic for analyzing the microbe's capability to break down proteins, provide bioactive compounds, and modify the texture and rheological properties of cream (Majeed et al. 2024). In the present study, all samples inoculated with BSN exhibited a noticeable rise in protease activity compared to the control, with sample 2 (incubated at 30°C for 24 h) showing the highest activity (Table 1). This suggests that the chosen temperature and incubation time provided optimal conditions for bacterial growth, metabolic activity, and enzyme synthesis, leading to the efficient utilization of nitrogen sources like ammonium and amino acids, and the enhancement of enzymatic secretion and stability.

Protease activity in the samples fermented under nonoptimal conditions was higher than that of the control, but the increase was not considerable. It signifies that BSN protease production is highly sensitive to the specific fermentation parameters such as temperature, time, and medium composition. These results are in line with the findings of Majeed et al. (2024), which stated that the production and activity of a thermostable protease from the *Bacillus subtilis BSP* strain are strongly influenced by incubation conditions. They particularly demonstrated that enzyme activity peaks under optimized conditions and declines when environmental parameters deviate from the optimum, which aligns with our observation that Sample 2 (incubated at 30°C for 24 h) exhibited the highest protease activity.

### 3.4 pH Measurement

pH is a key parameter reflecting biochemical changes in dairy products, influenced by bacterial activity and metabolism. Monitoring pH in cream indicates fermentation intensity, acid production, and microbial adaptation to process conditions (Seo 2023). In this research, the pH of all fermented cream samples with BSN showed a significant (*p* < 0.05) reduction compared to the control (Table 2), which was a sign of putting into action the acidogenic metabolic pathways as a result of bacterial inoculation. Among the treatments, Sample 2 (incubated at 30°C for 24 h) had the lowest pH, which means that this temperature–time combination provided the most favorable conditions for metabolic activity and acid production. The nonlinear trend of pH that was noticed during the prolonged incubation indicated that there was a metabolic equilibrium established between acid production and buffering or neutralization reactions.

**TABLE 2:** pH and acidity of creams inoculated with *Bacillus subtilis natto* and incubated in different temperatures and times.

Sample	pH	Acidity (%)
Control	6.51g*±0.0057	0.01660a±0.04158
Sample 1 (incubated in 30°C for 12 h)	4.36b±0.0115	0.007793d±0.01701
Sample 2 (incubated in 30°C for 24 h)	4.29a±0.0057	0.009200e±0.02707
Sample 3 (incubated in 30°C for 36 h)	4.38c±0.0057	0.007407d±0.02285
Sample 4 (incubated in 37°C for 12 h)	4.52e±0.0057	0.004987c±0.05227
Sample 5 (incubated in 37°C for 24 h)	4.47d±0.0115	0.005713c±0.03828
Sample 6 (incubated in 37°C for 36 h)	4.63f±0.0057	0.003963b±0.07769

* Different lowercase letters show differences between samples according to the Duncan test (*p* < 0.05).

At 37°C, although the pH of all inoculated samples remained lower than the control, higher temperature for a longer period appeared to alter the metabolic pathways of BSN, consequently limiting acid accumulation. Similar effects on pH depending on temperature and time have been observed in natto fermentation systems, where fermentation parameters greatly influence pH and acid production (Ma et al. 2025).

### 3.5 Acidity

Acidity is one of the major quality indicators in dairy cream, affecting the taste, physicochemical stability, shelf life, and sensory acceptability of the product directly. The alterations in acidity are indicative of the biological activity and the extent of the metabolic reactions occurring in the cream matrix during incubation (Seo 2023). The present research showed that the cream acidity was greatly influenced by the BSN inoculation and the changes in the incubation conditions (*p* < 0.05; Table 2). In contrast to the common expectations in many dairy fermentation systems, all inoculated samples were less acidic than the control, which means that the presence of BSN does not necessarily lead to increased accumulation of organic acids, and that acidity variations depend strongly on environmental characteristics and process conditions. A temperature of sample 2 (incubated at 30°C for 24 h) revealed higher acidity compared to other samples at the same temperature; however, the acidity was still less than the control. This points to a relative balance between the production and consumption of acidic compounds at this temperature.

In contrast, at 37°C an obvious decrease in acidity was observed with increasing incubation time, with sample 6 (incubated in 37°C for 36 h) showing the lowest acidity among inoculated treatments. These results imply that the metabolic activity of BSN at high temperatures could be in forms that limit the net production of acidic compounds, perhaps due to the shift of metabolic pathways or the availability of substrate in the high‐fat cream matrix. Such results demonstrate that the acidity of the fortified cream was more a function of the incubation temperature and duration rather than merely the presence of the bacterium and that the metabolic adaptation of BSN to the cream environment can not only reduce acidification but also enhance the sensory properties.

Although direct studies on acidity changes in dairy systems inoculated with BSN are rare, the studies related to the work in natto fermentation systems support the concept that fermentation parameters significantly influence the production of acidic components. For instance, the study on cofermentation of BSN with *Limosilactobacillus fermentum* demonstrated that pH and titratable acidity changed considerably with varying fermentation conditions, thus indicating that the interaction of microbes and the variables of the process determines acid production in natto‐type systems (Ma et al. 2025). These observations provide comparative evidence that the metabolic activity and fermentation dynamics of BSN can effectively influence acidity patterns, consistent with the nonlinear, condition‐dependent acidity changes that have been observed in the present cream matrix.

### 3.6 Measurement of Nattokinase Activity

Nattokinase activity serves as a major indicator of BSN activity in fermented products and is responsible for the physicochemical and biological properties of the matrix (Zheng et al. 2025). The present experiment showed that all samples inoculated with BSN exhibited a remarkable increase in nattokinase activity compared to the control, which had no detectable activity. Among them, Sample 2 (incubated at 30°C for 24 h) exhibited the highest nattokinase activity (Table 3), indicating that this particular temperature and incubation time were more suitable for microbial growth, metabolic activity, and enzyme synthesis.

**TABLE 3:** Nattokinase activity, MK‐4 and MK‐7 of creams inoculated with *Bacillus subtilis natto* and incubated in different temperatures and times.

Sample	Nattokinase activity (FU/g sample)	MK‐4 (ng/g)	MK‐7 (ng/g)
Control	0a	10.65a±0.3842	3.88a±0.18862
Sample 1 (incubated in 30°C for 12 h)	23.12f±0.2820^*^	89.91f±0.6747	21.39b±1.0945
Sample 2 (incubated in 30°C for 24 h)	32.87g±1.1389	140.35g±2.2950	44.61c±0.5888
Sample 3 (incubated in 30°C for 36 h)	20.81e±0.5441	81.66e±1.8267	22.99d±0.7026
Sample 4 (incubated in 37°C for 12 h)	13.69c±0.6624	59.49c±1.2903	11.21e±0.7016
Sample 5 (incubated in 37°C for 24 h)	18.91d±0.6022	65.79d±1.2113	13.21f±0.3716
Sample 6 (incubated in 37°C for 36 h)	9.38b±0.6037	37.65b±0.6492	7.34g±0.1195

^*^ Different lowercase letters show differences between samples according to the Duncan test (*p* < 0.05).

There are multiple reasons why the maximum enzyme activity was observed under optimal conditions. At 30°C and 24 h, BSN cells are presumably in their active growth phase, which has the highest metabolic flux directed towards the synthesis of extracellular enzymes, including nattokinase. The efficient use of nitrogen sources and the favorable pH–acidity conditions prevailing under the parameters also help to maintain enzymes' stability and promote their secretion. Samples incubated at nonoptimal conditions (higher temperature or shorter/longer times) exhibited lower activity, which is a clear reflection of the well‐known sensitivity of nattokinase production to fermentation parameters, indicating that stress or suboptimal metabolism can result in reduced enzyme yields (Yang et al. 2021).

In comparison to other fermented products, the nattokinase activity achieved in this study (32.87 FU/g) falls within the range of values reported for various products. However, there are previous reports with much higher nattokinase activity. For example, Yang and Yang (2025) reported a nattokinase activity of 124.79 IU/mL in fermented chestnut milk using BSN under optimized conditions (inoculum 4%, 48 h, 39°C, chestnut‐to‐milk ratio 1:3), which is approximately 3.8 times higher than our result. However, it should be noted that this study used a longer fermentation time (48 h vs. 24 h) and a higher temperature (39°C vs. 30°C). Another study by Zhang et al. (2021) reported a thrombolytic activity of 215.1 U/mL in fermented milk after only 8 h of fermentation with *Bacillus subtilis* JNFE0126, demonstrating that milk‐based substrates can support rapid nattokinase production.

### 3.7 Measurement of Menaquinone‐4 and Mk‐7 Content

Menaquinones (vitamin K2) are important bioactive compounds in fermented products, which have different biological properties and biosynthetic pathways depending on the length of the isoprenoid chain (Kang et al. 2022). In dairy products, the concentration of MK‐4 in dairy products is typically low, and its origin is mostly assigned to metabolic transformations in the animal body; however, the outcome of this research indicated that cream fermentation with BSN could significantly (*p* < 0.05) increase both MK‐4 and MK‐7 isoforms (Table 3).

**FIGURE 1:**
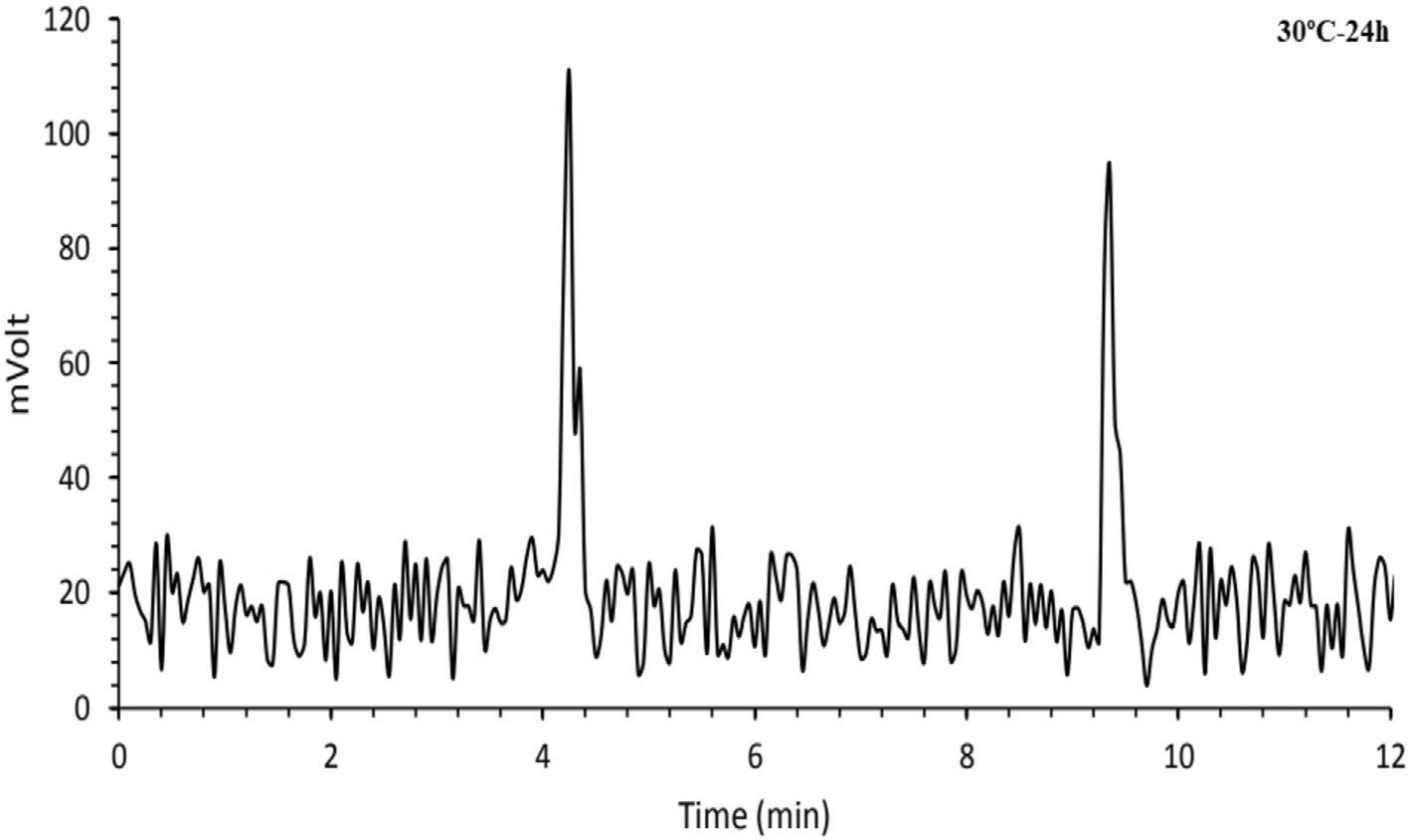
HPLC chromatogram showing MK‐4 and MK‐7 peaks, respectively, in fermented cream sample inoculated with *Bacillus subtilis natto* at 30°C for 24 h.

The simultaneous examination of MK‐4 and MK‐7 indicated that all treated samples with inoculum showed a considerable increase in both vitamins compared to the control sample, although the trends for the two compounds were not entirely alike. Under all fermentation conditions, MK‐4 was in absolute numbers higher than MK‐7, which is possibly due to the metabolic preference of this bacterium for producing shorter‐chain isoforms in dairy matrices. Nevertheless, the two vitamins had the highest levels in sample 2 (incubated at 30°C for 24 h), which might point to a shared optimum for the functioning of the vitamin K2 biosynthetic pathways (Figure 1).

From a biochemical perspective, the simultaneous increase in MK‐4 and MK‐7 under optimal conditions indicates that the isoprenoid synthesis pathway and key enzymes such as MenA and MenG were more efficient at 30°C and moderate fermentation time. The situation described a proper ratio is maintained between cell growth, energy supply, and enzyme stability, and the attachment of isoprenoid precursors to the vitamin K2 side chains is done efficiently. At lower temperatures, metabolic limitation and a decrease in bacterial growth rate prevent vitamin accumulation, while at 37°C, although growth is possible, heat stress and reduced enzyme stability cause a decrease in the production of both isoforms (Yuan et al. 2020).

The impact of fermentation time on MK‐4 and MK‐7 showed that increasing the time from 12 to 24 h significantly raised their levels, while extending fermentation to 36 h resulted in a decline. This decrease is likely due to bacteria entering the stationary phase, reconsumption of vitamins in metabolic reactions, and potential oxidative degradation of sensitive compounds. Overall, although MK‐4 was higher in all samples, the relative increase of MK‐7 was more pronounced, emphasizing the role of BSN fermentation not only in absolute vitamin accumulation but also in enhancing the nutritional value of the product, particularly with vitamins that have a longer half‐life, such as MK‐7 (Bonaldo and Leroy 2024).

Compared to other fermented dairy products, the MK‐7 level achieved in this study (43.93 ng/g) is similar to that of kefir (48.2 ng/g) and significantly higher than that of probiotic yogurt (17.0 ng/g) as reported by Altuncu et al. (2024). Additionally, Fu et al. (2017) found that conventional full‐fat yogurt and cheese had undetectable levels of MK‐7, with only trace amounts of MK‐4 (≈7 ng/g) present. These comparisons demonstrate that using BSN in cream fermentation results in significantly higher levels of vitamin K2 compared to conventional dairy starter cultures.

The observed increase in MK‐7 production due to BSN is supported by previous studies on bacterial vitamin K2 biosynthesis. Strains isolated from traditional natto are known to efficiently produce MK‐7 under optimized fermentation conditions, and strategies such as medium optimization and static culture have been reported to significantly enhance MK‐7 yields in natto fermentation systems (Kang et al. 2022).

### 3.8 Total Microbial Count

**FIGURE 2:**
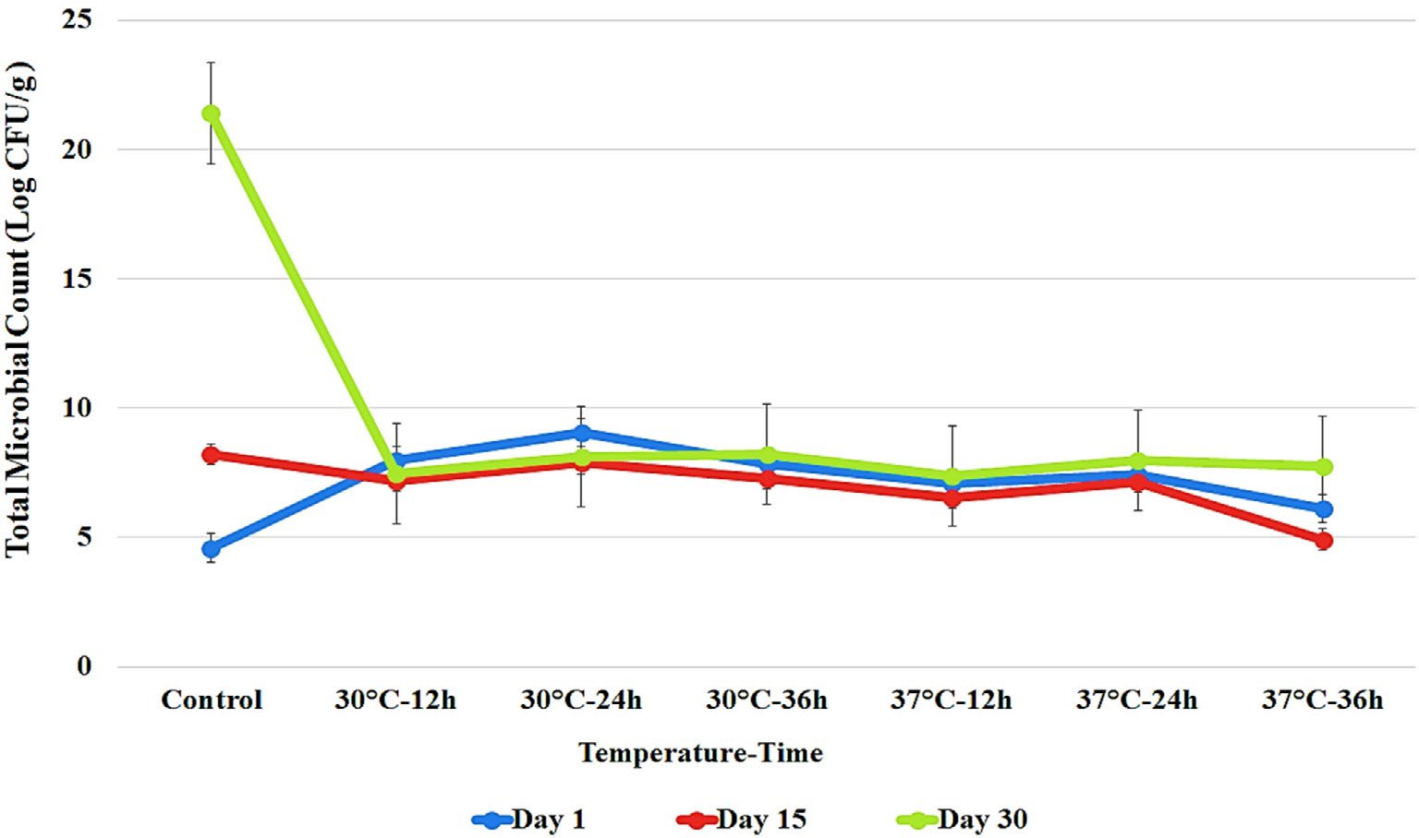
Changes in total microbial count of pasteurized cream samples inoculated with *Bacillus subtilis natto* during storage.

Total microbial count is a critical sign of microbiological safety and the shelf‐life period of dairy products. The control sample in this study exhibited the highest microbial load during storage, particularly on day 30, which points to the presence of spoilage microorganisms in the absence of fermentation. Conversely, all samples inoculated with BSN showed significantly (*p* < 0.05) lower microbial counts throughout storage (Figure 2), thus a clear inhibitory effect of the fermentation process on undesirable microflora was demonstrated. Among the treatments, sample 2 (incubated at 30°C for 24 h) exhibited a lower microbial load, which implied that this combination of temperature and time provides optimal conditions for microbial control and product safety (Figure 2).

The inhibitory effect that was noted in the fermented samples is linked to the activity of the BSN, which produces antimicrobial compounds such as poly‐γ‐glutamic acid, bacteriocins, and antibacterial peptides. These metabolites inhibit spoilage of microorganisms by disrupting cell membranes, interfering with metabolic pathways, and limiting nutrient availability (Lin et al. 2021). A comparable antimicrobial behavior has already been noted in natto‐based dairy fermentation systems, where the application of BSN significantly reduced total microbial counts and suppressed competing microflora, thereby improving microbiological stability and extending shelf life. The minor rise in the number of microorganisms observed in some treatments at the end of the storage period could be attributed to the decline in bacterial metabolism, the breakdown of the antimicrobial compounds, or the conditions of storage and packaging. Nevertheless, the total findings clearly demonstrate that BSN fermentation can be an efficient way to improve the microbial safety and stability of cream over time. These findings are in line with previous studies on natto fermentation systems, which point out that optimized fermentation conditions lead to the highest antimicrobial metabolite production and help maintain microbiological quality during storage (Afzaal et al. 2022).

### 3.9 Sensory Evaluation

**FIGURE 3:**
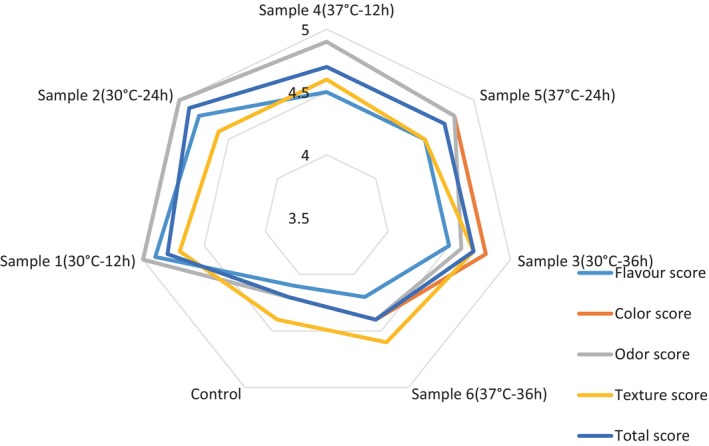
Sensory evaluation chart for different samples of pasteurized cream samples inoculated with *Bacillus subtilis natto* on different days of storage.

The analysis of sensory evaluation results showed that the addition of BSN to pasteurized cream not only did not cause a decrease in product quality, but also improved sensory properties in many aspects. The fermentation conditions, particularly with a temperature of 30°C and a time of 12 and 24 h, obtained higher scores for taste, smell, and texture compared to the control sample (Figure 3). This implies that controlled fermentation can prevent the development of off‐flavors caused by fat oxidation or increased microbial load in the control sample by producing desirable flavor compounds such as mild organic acids and diacetyl. The enhancement of texture uniformity, proper consistency, and pleasant mouthfeel might also be due to the production of biopolymers such as γ‐polyglutamic acid produced through the activity of this bacterium.

As far as the smell is concerned, it was noticed that the fermentation treatments had a less strong and more pleasant aroma compared to the control sample. This points out the important role of fermentation in preventing the development of unpleasant odors resulting from the growth of undesirable microorganisms during storage. On the other hand, the color and appearance of the samples were examined, and it was revealed that the presence of bacteria did not have a negative effect on the color of the cream; even in some treatments, the uniformity of the product structure was greater, which led to better acceptance by the evaluators.

From the overall findings, it was concluded that the best fermentation conditions were 30°C and 24 h, which was the most appropriate treatment from the sensory viewpoint and got the highest overall score. In contrast, the control sample, which did not contain bacteria, got the lowest scores, clearly indicating the considerable role that BSN played in suppressing oxidative spoilage and improving the appearance and organizational characteristics of cream. The results show overall that the utilization of this bacterium can lead to the production of a product with a more desirable sensory quality than traditional pasteurized cream, and in this regard, it is considered an advantageous option for the development of functional and probiotic dairy products (Foguel et al. 2021).

## 4 Conclusion

The application of BSN in the production of fermented products revealed that this approach can be applied to produce dairy products with a high content of vitamin K2. The fermentation with this bacterium enhanced cream stability and texture, particularly at 30°C. In addition, microbial analysis confirmed safe total microbial loads, with BSN dominating and inhibiting contaminants. Sensory evaluations revealed that samples fermented 12–24 h at 30°C had the highest score for taste, aroma, and overall acceptance. To sum up, BSN improved the functional, sensory, and health‐related aspects of cream, offering a promising approach for new vitamin K2 and nattokinase‐enriched dairy products. Also, there is a need for further research to study the potential synergistic effects of combining BSN with conventional dairy starter cultures, as well as investigating the potential health benefits of the finished product.

## Author Contributions

**Sodeif Azadmard‐Damirchi:** conceptualization, writing – review and editing, supervision, project administration, validation. **Hasan Moharampour:** formal analysis, investigation, writing – original draft, data curation. **Safar Farjnia:** methodology, validation, visualization. **Javad Hesari:** methodology, supervision, writing – review and editing. **Mohammad Reza Afshar Mogaddam:** formal analysis, methodology.

## Funding

This research was funded by the University of Tabriz (grant no. D‐13‐5502 and D‐6588‐13).

## Disclosure

Author approval and responsibility statement: All authors have read and approved the final version of the manuscript. Sodeif Azadmard‐Damirchi had full access to all of the data in this study and takes complete responsibility for the integrity of the data and the accuracy of the data analysis.

## Ethics Statement

Ethical approval was not required for this study because it involved only physicochemical, microbiological, and sensory analyses of a fermented dairy product with no human or animal subjects. Sensory evaluation was performed by trained panelists on an anonymized voluntary basis and informed consent and did not involve any clinical or biological sampling.

## Conflicts of Interest

The authors declare no conflicts of interest.

## Data Availability

The data that support the findings of this study are available from the corresponding author upon reasonable request.
